# Evaluation of the clinical frailty scale for predicting mortality or functional dependence at ICU discharge: A cohort study

**DOI:** 10.1038/s41598-025-23243-0

**Published:** 2025-11-12

**Authors:** Amanda Christina Kozesinski-Nakatani, Rafaella Stradiotto Bernardelli, Jeane Cristina Fonseca Vieira, Marcelo José Martins-Junior, Maria Nesryn Tiba, Verônica Silva Barros, Taher Tiba, Beatriz Lottermann Konzen, Kamila Janiscki, Álvaro Réa-Neto, Auristela Duarte de Lima Moser

**Affiliations:** 1 Pontifical Catholic University of Paraná (PUCPR), Curitiba, PR Brazil; 2 Center for Studies and Research in Intensive Care (CEPETI), Curitiba, PR Brazil; 3 Hospital Santa Casa de Curitiba, Curitiba, PR Brazil

**Keywords:** Risk factors, Epidemiology, Epidemiology

## Abstract

Frailty is a critical factor influencing outcomes in intensive care units (ICUs). A retrospective cohort study involving 8041 adult ICU patients in Curitiba (May 2023-April 2024) evaluated the role of the Clinical Frailty Scale (CFS) as a mortality risk and functional dependence at ICU discharge. Patients were classified according to their CFS score at admission, with higher scores indicating greater frailty. The primary outcomes were ICU mortality and transition to a higher level of functional dependence at discharge. Multivariable models were adjusted for age, sex, number of comorbidities, type of hospitalization, presence of limitations on life-sustaining therapies, and SOFA score within the first 24 h. The cohort had a mean age of 66 years (51.4% women), with a median SOFA score of 2. Most patients were classified as “managing well” (33.5%), “vulnerable” (21%), or “mildly frail” (10.1%). Overall ICU mortality was 11.5%, and more than 40% of patients were discharged with some degree of functional dependence. Higher frailty at admission was independently associated with worse outcomes, including increased mortality and greater functional dependence. These findings reinforce the value of frailty assessment by using the CFS to support prognostic decision-making in critically ill patients.

**Supplementary Information:**

The online version contains supplementary material available at 10.1038/s41598-025-23243-0.

## Introduction

The intensive care unit (ICU) is a specialized environment where critically ill patients receive high quality care with close observation^1^. The mortality rates in the ICU vary depending on the underlying condition of the patient and are generally higher than those in other hospital units^2^. In Brazil, the mortality rate for patients in the ICU is about 18%^3^, highlighting the importance of predicting the prognosis of these patients. For this purpose, predictive scales can be used to identify patients at greater risk of deterioration and allow for faster and more effective interventions^4,5^.

The Clinical Frailty Scale (CFS) assesses the vulnerability of an individual (especially older ones) to adverse health events. It evaluates specific domains—including comorbidity, functional status, and cognition—to generate a frailty score that ranges from 1 (very fit) to 9 (terminally ill)^6^. Initially created to evaluate a patient’s functional deterioration and physical capacity, the scale assesses multiple dimensions of health, providing an overview of the individual’s overall status^7,8^. As a result, the clinical use of this scale has expanded over the years, and the scale has even been applied in the ICU setting for prognosis evaluation^9^.

The CFS stands out in this scenario as a complementary tool for predicting the level of functional dependence and mortality in patients admitted to the ICU^10^. Patients classified as frail (i.e., those with high CFS scores) exhibit higher mortality rates both during hospitalization and in the long term, potentially due to sarcopenia and neuromuscular weakness^11–13^.

Based on these considerations, the present study aims to carefully assess the prognostic value of the CFS in predicting both mortality and long-term functional dependence among patients admitted to the Intensive Care Unit.

## Methods

This historical cohort study included patients aged 18 years or older who were consecutively admitted to ICUs across five hospitals in Curitiba (Brazil) between May 1, 2023, and April 30, 2024. The study was conducted across 123 ICU beds, encompassing both the public and private health systems.

The study was approved by the Ethics Committee of the Instituto de Neurologia de Curitiba under protocol 3,000,353 on September 03, 2018, CAAE 98099918.2.0000.5227 and the need for informed consent was waived due to the noninterventional study design and data collection (we only reviewed medical records without contacting the participants). All research procedures were conducted in accordance with the ethical guidelines outlined in Resolution 466/2012 of the National Health Council (NHC) for research involving human beings and the Declaration of Helsinki (8th revision, 2024).

Patients’ data were obtained from the database of the Center for Studies and Research in Intensive Care Medicine (CEPETI). This database has a clinical and care-oriented nature and serves, secondarily, a research purpose. Intensive care physicians and residents who work in the ICUs affiliated with CEPETI update the database daily with clinical and epidemiological data on the admission and discharge of all hospitalized patients. Importantly, the variables selected a priori for this study are entered into structured, closed, and mandatory fields of the database, which ensures systematic recording and prevents missing data for these variables.

All hospitalizations of patients aged over 18 years, of both sexes, and occurring within the pre-established data collection periods were considered in the present study. The study excluded data from duplicate medical records, patients transferred to ICUs not affiliated with CEPETI, admissions due to trauma, pregnant women, patients transferred from other health services, and cases in which data on age, reason for admission, or ICU outcome were not reported.

Frailty was assessed at the time of ICU admission using the CFS, which is based on questions about the patient’s conditions 2 weeks prior to the admission. The patients’ frailty status was scored on an ordinal scale of 1 to 9 according to the following CFS scores: 1, very fit; 2, well; 3, managing well; 4, vulnerable; 5, mildly frail; 6, moderately frail; 7, severely frail; 8, very severely frail; and 9, terminally ill. The analyses were carried out considering the nine classification levels of the CFS scale (A) separately, (B) grouped into three levels according to CFS score (no frailty [scores 1–4], mild/moderate frailty [scores 5–6], and severe frailty [scores 7–9]) and (C) dichotomized into two categories according to CFS score (no frailty [scores 1–4] and frailty [scores 5–9]).

In addition to the frailty status (exposure variable), data collected from the patients included in the analysis also covered age; sex; comorbidities; whether or not the patient received care in the public health sector; reason for ICU admission; type of ICU admission (if medical, emergency surgical, or elective surgical); requirement for hemodynamic support with vasoactive drugs, invasive ventilatory support, or renal replacement therapy during ICU admission; Acute Physiology and Chronic Health Evaluation II (APACHE II) severity score in the first 24 h in the ICU; Sequential Organ Failure Assessment (SOFA) score in the first 24 h in the ICU; implementation and level of limitation of life-sustaining treatment; duration of ICU stay; ICU mortality; and level of functional dependence according to the intensive care physician at the time of ICU discharge for patients discharged due to clinical improvement, assessed on a four-level scale (fully independent, dependent on assistance with complex activities (e.g., cooking, cleaning, driving), dependent on assistance with basic activities (e.g., bathing, dressing, eating), and dependent on assistance with all activities). The variable selection rationale is detailed in the Supplementary Material.

The primary outcome was the highest level on a scale of functional dependence and mortality at ICU, categorized as follows: 1 (fully independent), 2 (dependent on assistance with complex activities), 3 (dependent on assistance with basic activities), 4 (dependent on assistance with all activities), and 5 (death). The two secondary outcomes were ICU mortality and the level of functional dependence at ICU discharge (i.e., dependence on assistance with basic activities or all activities at ICU discharge).

### Statistical analysis

Categorical variables are presented as numbers and percentages. Quantitative variables with normal distribution are presented as mean ± standard deviation values, while those without normal distribution are presented as median (interquartile range) values.

Associations between non-dichotomous categorical variables were analyzed using the chi-square test, followed by a row-by-row comparison of case proportions using the Z test, with the significance value adjusted by the Bonferroni method. Normally distributed quantitative variables among three or more groups were compared using one-way analysis of variance (ANOVA), followed by *post hoc* analysis with the Bonferroni test. When the assumption of normality was not met, the nonparametric Kruskal-Wallis test was used, followed by *post hoc* analysis using Dunn’s test with a Bonferroni-adjusted significance level.

Univariable and multivariable generalized linear models with ordinal logistic regression for the dependent variable, using hybrid parameter estimation and a fixed-value scale, were fitted to assess the association between frailty (on a nine-level ordinal scale, where higher values indicate worse frailty) and level of functional dependence at ICU discharge or death, classified on a five-point ordinal scale (where higher values also indicate a worse outcome). Additionally, univariable and multivariable generalized linear models with binary logistic regression for the dependent variable, using hybrid parameter estimation and a fixed-value scale, were fitted in the subgroup of patients discharged alive from the ICU to assess the association between frailty (on a nine-level ordinal scale, where higher values indicate worse frailty) and the presence of greater functional dependence at ICU discharge. The results of both models were expressed as odds ratios (ORs) with their respective 95% confidence intervals (CIs), and their statistical significance was determined using the Wald test.

Univariable and multivariable Cox regression models were fitted to assess the influence of frailty (scored on a nine-level ordinal scale, with higher scores indicating worse frailty) on the instantaneous risk of death in the ICU. The results were expressed as hazard ratios (HRs) and 95% CIs, with the statistical significance determined by the Wald test. The proportional hazards assumption was confirmed by a graphical analysis of scaled Schoenfeld.

All multivariable models were adjusted for variables obtained at ICU admission, which were selected a priori based on a literature review. These variables included age^13–15^, female sex (compared with male sex)^14,15^, number of comorbidities (one, two, or three compared with none)^14^, type of hospitalization (medical and emergency surgical compared with elective surgical)^16^, presence of some level of limitation of life-sustaining treatment at admission (compared with no limitation), and SOFA score in the first 24 h in the ICU.

Sensitivity analyses were conducted for all models considering CFS scores categorized as follows: (A) into three levels (no frailty [scores 1–4], mild/moderate frailty^5]– [6^, and severe frailty^7–9^ and (B) dichotomized into two categories (no frailty [scores 1–4] and frailty [scores 5–9]).

The level of statistical significance was set at 5%, and the data were analyzed using the statistical software IBM SPSS Statistics, version 29.0 (IBM SPSS Inc., Chicago, IL, USA). Missing data was not imputed.

## Results

**Fig. 1:**
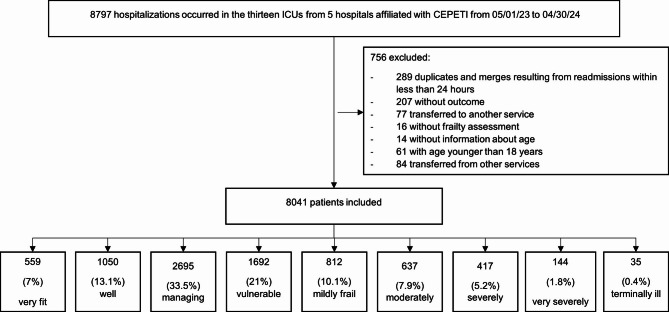
Sampling flowchart.

Between May 1, 2023, and April 30, 2024, a total of 8797 hospitalizations were recorded in the ICUs of the five participating hospitals. Of these, 756 were excluded based on the selection criteria. Thus, 8041 hospitalizations made up the sample of this study (Fig. 1). In the final cohort of 8,041 patients, none of the variables analyzed in this study presented missing data, as they were recorded in mandatory structured fields of the CEPETI database.

The main causes of hospitalization were cardiovascular diseases (38.5%), followed by respiratory (13.9%) and neurological (13.9%) conditions (Table S1). These results confirm a high prevalence of comorbidities related to the cardiovascular system among the critically ill patients analyzed. At the time of admission, 16.1% of patients required invasive ventilation, while 18.7% required hemodynamic support with vasoactive drugs (Table S1). These data reflect the severity of the cases and the need for critical interventions throughout the hospitalization. Detailed characteristics of the sample are presented in Table 1.

**Table 1:** Characteristics of the study sample and comparison of the patients’ characteristics at hospitalization and outcomes, with patients categorized according to the nine levels of the clinical frailty Scale.

Variables	Total (*n* = 8041)	1 - Very fit (*n* = 559)	2 - Well (*n* = 1050)	3 - Managing well (*n* = 2695)	4 - Vulnerable (*n* = 1692)	5 - Mildly frail (*n* = 812)	6 - Moderately frail (*n* = 637)	7 - Severely frail (*n* = 417)	8 - Very severely frail (*n* = 144)	9 - Terminally ill (*n* = 35)	*P* value
Age	66 ± 17.3	54.2 ± 18 _a_	55.7 ± 18.6 _a, b_	63.3 ± 15.9 _c_	68 ± 14.1 _d_	74.1 ± 13.4 _e_	77.5 ± 13.3 _e, f_	76.7 ± 15.8 _g, f_	83.1 ± 12.5 _h_	77.7 ± 15.8 _e, f,g_	< 0.001^a^
Female Sex	4131 (51.4%)	264 (47.2%) _a_	485 (46.2%) _a_	1321 (49%) _a_	863 (51%) _a, b_	463 (57%) _b, c_	362 (56.8%) _b, c_	257 (61.6%) _c_	97 (67.4%) _c_	19 (54.3%) _a, b,c_	< 0.001^c^
Number of comorbidities											< 0.001^c^
None	1487 (18.5%)	201 (36%) _a_	331 (31.5%) _a_	612 (22.7%) _b_	210 (12.4%) _c_	71 (8.7%) _c, d_	40 (6.3%) _d_	17 (4.1%) _d_	5 (3.5%) _d_	0 (0%) _c, d_	
One	2415 (30%)	183 (32.7%) _a, b_	348 (33.1%) _a, b_	879 (32.6%) _a, b_	509 (30.1%) _a, b_	177 (21.8%) _c_	167 (26.2%) _a, b,c_	104 (24.9%) _a, b,c_	31 (21.5%) _b, c_	17 (48.6%) _a_	
Two	2154 (26.8%)	117 (20.9%) _a_	229 (21.8%) _a_	703 (26.1%) _a, b_	490 (29%) _b, c_	265 (32.6%) _c_	181 (28.4%) _a, b,c_	117 (28.1%) _a, b,c_	44 (30.6%) _a, b,c_	8 (22.9%) _a, b,c_	
Three or more	1985 (24.7%)	58 (10.4%) _a_	142 (13.5%) _a, b_	501 (18.6%) _c_	483 (28.5%) _d_	299 (36.8%) _e_	249 (39.1%) _e_	179 (42.9%) _e_	64 (44.4%) _e_	10 (28.6%) _b, c,d, e_	
Type of hospitalization											< 0.001^c^
Elective surgical	2423 (30.1%)	148 (26.5%) _a, b_	332 (31.6%) _b, c_	1038 (38.5%) _d_	585 (34.6%) _c, d_	196 (24.1%) _a_	82 (12.9%) _e_	36 (8.6%) _e, f_	5 (3.5%) _f_	1 (2.9%) _a, e,f_	
Emergency surgical	713 (8.9%)	40 (7.2%) _a_	109 (10.4%) _a_	235 (8.7%) _a_	160 (9.5%) _a_	75 (9.2%) _a_	63 (9.9%) _a_	27 (6.5%) _a_	4 (2.8%) _a_	0 (0%) _a_	
Medical	4905 (61%)	371 (66.4%) _a_	609 (58%) _b_	1422 (52.8%) _b_	947 (56%) _b_	541 (66.6%) _a_	492 (77.2%) _b_	354 (84.9%) _c, d_	135 (93.8%) _d_	34 (97.1%) _c, d_	
LLST on admission											‘
A	7670 (95.4%)	557 (99.6%)	1042 (99.2%)	2667 (99%)	1657 (97.9%)	777 (95.7%)	569 (89.3%)	322 (77.2%)	71 (49.3%)	8 (22.9%)	
B	306 (3.8%)	2 (0.4%)	6 (0.6%)	22 (0.8%)	33 (2%)	32 (3.9%)	67 (10.5%)	79 (18.9%)	49 (34%)	16 (45.7%)	
C	65 (0.8%)	0 (0%)	2 (0.2%)	6 (0.2%)	2 (0.1%)	3 (0.4%)	1 (0.2%)	16 (3.8%)	24 (16.7%)	11 (31.4%)	
APACHE II in the first 24 h	11 (7–17)	8 (4–11) _a_	8 (5–12) _a, b_	10 (6–14) _c_	12 (8–18) _d_	15 (10–21) _e_	16 (12–23) _e, f_	18 (14–25) _g_	23 (18–31.5) _h_	22 (17–30) _f, g,h_	< 0.001^#^
SOFA score in the first 24 h	2 (0–4)	1 (0–2) _a_	1 (0–3) _a, b_	1 (0–4) _c_	2 (1–4.5) _d_	3 (1–5) _e_	3 (2–6) _e, f_	4 (2–7) _g_	5 (4–8) _h_	5 (4–7) _g, h_	< 0.001^#^
Length of ICU stay (days)	2 (1–4)	2 (1–3) _a_	2 (1–3) _a, b_	2 (1–3) _b, c_	2 (1–5) _d_	3 (2–6) _e_	3 (2–6) _f_	4 (2–7) _f, g_	4 (2–7) _e, f,g, h_	2 (1–5) _a, b,c, d,e, f,h_	< 0.001^#^
Mortality	927 (11.5%)	19 (3.4%) _a_	50 (4.8%) _a_	151 (5.6%) _a_	211 (12.5%) _b_	142 (17.5%) _c_	141 (22.1%) _c, d_	116 (27.8%) _d_	75 (52.1%) _e_	22 (62.9%) _e_	< 0.001^*^
Level of functional dependence at discharge											< 0.001^*^
Fully independent	2903 (36.1%)	400 (71.6%) _a_	614 (58.5%) _b_	1176 (43.6%) _c_	503 (29.7%) _d_	147 (18.1%) _e_	52 (8.2%) _f_	10 (2.4%) _g_	1 (0.7%) _g_	0 (0%) _e, e,g_	
Dependent on assistance with complex activities	2745 (34.1%)	111 (19.9%) _a_	322 (30.7%) _b_	1053 (39.1%) _c_	712 (42.1%) _c_	334 (41.1%) _c_	180 (28.3%) _b_	30 (7.2%) _d_	3 (2.1%) _d_	0 (0%) _a, d_	
Dependent on assistance with basic activities	1033 (12.8%)	21 (3.8%) _a_	57 (5.4%) _a_	287 (10.6%) _b_	225 (13.3%) _b, c_	134 (16.5%) _c_	183 (28.7%) _d_	117 (28.1%) _d_	8 (5.6%) _a, b_	1 (2.9%) _a, b,c_	
Dependent on assistance with all activities	433 (5.4%)	8 (1.4%) _a, b_	7 (0.7%) _b_	28 (1%) _b_	41 (2.4%) _a_	55 (6.8%) _c_	81 (12.7%) _d_	144 (34.5%) _e_	57 (39.6%) _e_	12 (34.3%) _e_	
Death	927 (11.5%)	19 (3.4%) _a_	50 (4.8%) _a_	151 (5.6%) _a_	211 (12.5%) _b_	142 (17.5%) _c_	141 (22.1%) _c, d_	116 (27.8%) _d_	75 (52.1%) _e_	22 (62.9%) _e_	

Age is reported as mean ± standard deviation, while the other quantitative variables are reported as mean or median (interquartile range). Categorical variables are described as frequency (percentage). The categories of LLST included the following: A, all necessary measures; B, all necessary measures but not including cardiopulmonary resuscitation; C, withholding of support; D, withdrawal of support; E, brain death. ^§^ Statistical significance determined by one-way ANOVA followed by *post hoc* analysis with the Bonferroni test. ^#^ Statistical significance determined by the nonparametric Kruskal-Wallis test followed by *post hoc* analysis using Dunn’s test with a Bonferroni-adjusted significance level. ^*^ Statistical significance determined by the chi-square test followed by a row-by-row comparison of case proportions using the Z test, with the significance value adjusted by the Bonferroni method. -’ The chi-square test could not be applied due to some cells with an expected frequency lower than 1. Subscript letters: The results of two-by-two comparisons (post hoc) are expressed in letters, in which equal letters indicate that there is no significant difference between the groups and different letters indicate that there is significant difference (*p* < 0.05). Abbreviations: APACHE II, Acute Physiology and Chronic Health Evaluation II; ICU, intensive care unit; SOFA, Sequential Organ Failure Assessment.

The median CFS score in the sample was 3 (IQR 3–5), indicating that most patients had either no frailty or mild-to-moderate frailty. Among the different CFS levels, 33.5% of patients were classified as “managing well,” 21% as “vulnerable,” and 10.1% as “mildly frail” (Table 1). Table 1 also presents a detailed comparison of the patients’ characteristics on admission and outcomes across the nine CFS levels, demonstrating a significant association between CFS scores with ICU mortality and with the level of functional dependence upon ICU discharge.

The overall ICU mortality was 11.5%, with 927 deaths recorded and more than 40% of patients who survived presenting some degree of functional dependence (Table 1). An analysis of outcomes by level of frailty demonstrated a relationship between greater frailty and increased mortality risk: 62.9% of patients classified as “terminally ill” died, as did 27.8% of those considered “severely frail.” In contrast, patients with reduced frailty, including those categorized as “very fit,” had reduced mortality, reinforcing the correlation between reduced frailty and improved prognosis (Table 1).

In the univariable analysis, patients classified as “vulnerable,” “mildly frail,” “moderately frail,” “severely frail,” “very severely frail,” or “terminally ill,” compared with those classified as “very fit,” had greater cumulative odds of death or a worse clinical condition at ICU discharge, according to the five-point ordinal scale adopted. Likewise, the variables selected a priori to adjust the relationship between frailty and ICU outcome were individually associated with worse ICU outcomes (Table S2, Figure S1).

**Fig. 2:**
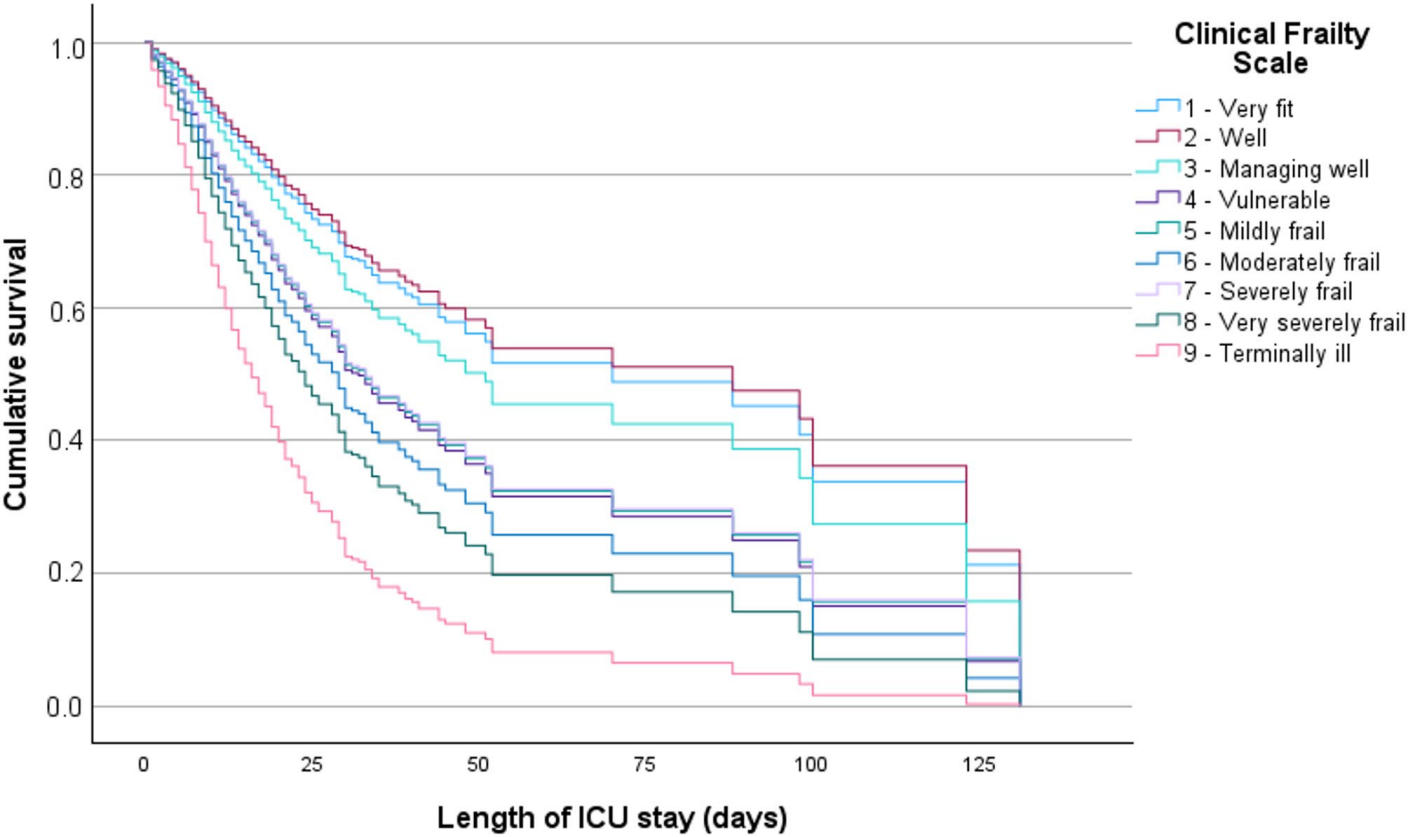
**Comparison of the nine Clinical Frailty Scale levels in relation to the instantaneous risk of death in the ICU.** The results are presented in cumulative survival over time for each of the groups as a result of the multivariable Cox regression model for predicting mortality in the ICU, adjusted for sex, age, number of comorbidities, type of hospitalization, and presence of limitation of therapeutic support at ICU admission.

A model adjusted for age, sex, number of comorbidities, type of hospitalization, limitation of life-sustaining treatment at ICU admission, and SOFA score in the first 24 h revealed that the greater the frailty status prior to ICU admission (i.e., higher CFS scores, relative to the lowest score [1 - very fit]), the greater the cumulative odds of death or a worse clinical condition at ICU discharge (Table 2). Furthermore, being admitted to the ICU with a status of “vulnerable,” “mildly frail,” “moderately frail,” “severely frail,” “very severely frail,” or “terminally ill” increased the instantaneous risk of death in the ICU, when compared with the status of “very fit,” independent from age, sex, number of comorbidities, type of hospitalization, organ dysfunctions in the first 24 h, and limitation of life-sustaining treatment at admission. In contrast, the statuses of “well” and “managing well” did not differ significantly from the status of “very fit” in relation to the risk of death (Table 3; Fig. 2).

**Table 2:** Multivariable generalized linear model with an ordinal logistic distribution assessing the likelihood of transition to a higher level of functional dependence upon ICU discharge or death.

Variables	Adjusted OR (95% CI) for greater level of functional dependence at ICU discharge or death ^a^	*P* value ^b^
Clinical Frailty Scale levels		
1 - Very fit	ref	
2 - Well	1.697 (1.352–2.129)	< 0.001
3 - Managing well	2.492 (2.029–3.061)	< 0.001
4 - Vulnerable	3.993 (3.221–4.95)	< 0.001
5 - Mildly frail	5.806 (4.577–7.364)	< 0.001
6 - Moderately frail	10.899 (8.493–13.988)	< 0.001
7 - Severely frail	20.081 (15.321–26.32)	< 0.001
8 - Very severely frail	27.735 (18.776–40.969)	< 0.001
9 - Terminally ill	38.666 (18.983–78.756)	< 0.001
Sex		
Male	ref	
Female	1.291 (1.187–1.406)	< 0.001
Age	1.017 (1.014–1.02)	< 0.001
Number of comorbidities		
None	ref	
One	1.034 (0.909–1.177)	0.607
Two	1.025 (0.897–1.173)	0.714
Three or more	1.034 (0.909–1.177)	0.607
Type of hospitalization		
Elective surgical	ref	
Emergency surgical	1.365 (1.164–1.600)	< 0.001
Medical	1.039 (0.943–1.145)	0.437
LLST at admission		
None	ref	
Some	1.853 (1.498–2.291)	< 0.001
SOFA score in the first 24 h	1.276 (1.257–1.296)	< 0.001
Number of cases in the model	8041	
Bayesian information criterion (BIC)	18127.453	

^a^ Odds ratio (95% confidence interval) from the multivariable generalized linear model with an ordinal logistic distribution of the dependent variable, using hybrid parameter estimation and fixed-value scale, for predicting the likelihood of transition to a higher level on the functional dependence upon ICU discharge or death (1: Fully independent; 2: Dependent on assistance with complex activities; 3: Dependent on assistance with basic activities; 4: Dependent on assistance with all activities; and 5: Death).

^b^ Significance level of the Wald test.

^c^ Bayesian information criterion (BIC) of the multivariate generalized linear model that represents the quality of the model given its explanatory potential; the lower the BIC, the more the model is able to explain the dependent variable.

**Table 3:** Multivariable Cox model for predicting the instantaneous risk of death in the ICU, considering the clinical frailty scale grouped into three levels.

Variables	Adjusted HR (95% CI) for ICU mortality ^a^	*P* value ^b^
Clinical Frailty Scale levels		
1 - Very fit	ref	-
2 - Well	0.937 (0.549–1.597)	0.810
3 - Managing well	1.194 (0.738–1.931)	0.471
4 - Vulnerable	1.746 (1.084–2.812)	0.022
5 - Mildly frail	1.706 (1.046–2.783)	0.032
6 - Moderately frail	2.051 (1.254–3.356)	0.004
7 - Severely frail	1.691 (1.026–2.788)	0.039
8 - Very severely frail	2.456 (1.441–4.185)	< 0.001
9 - Terminally ill	3.817 (1.99–7.323)	< 0.001
Sex		
Male	ref	-
Female	1.178 (1.033–1.344)	0.015
Age	1.011 (1.005–1.016)	< 0.001
Number of comorbidities		
None	ref	-
One	1.069 (0.816–1.399)	0.629
Two	0.969 (0.741–1.268)	0.819
Three or more	0.920 (0.704–1.201)	0.539
Type of hospitalization		
Elective surgical	ref	-
Emergency surgical	1.844 (1.385–2.455)	< 0.001
Medical	1.553 (1.231–1.96)	< 0.001
LLST on admission		
None	ref	-
Some	2.184 (1.767–2.700)	< 0.001
SOFA score in the first 24 h	1.184 (1.165–1.203)	< 0.001
Number of cases in the model	8041	

^a^ Hazard ratio (95% confidence interval) from the multivariable Cox regression model for predicting ICU mortality.

^b^ Significance level of the Wald test.

Abbreviations: HR, hazard ratio; ICU, intensive care unit; LLST, limitation of life-sustaining treatment; ref, reference; SOFA, Sequential Organ Failure Assessment.

In an analysis focused exclusively on patients discharged alive from the ICU, those classified upon ICU admission as “managing well,” “vulnerable,” “mildly frail,” “moderately frail,” “severely frail,” or “very severely frail,” relative to those classified as “very fit,” had significantly greater odds of being discharged from the ICU depending on assistance with basic activities or all activities, both in the unadjusted and adjusted models. Only individuals classified as “well” did not differ significantly from those classified as “very fit” in terms of the odds of both these outcomes (Table 4).

**Table 4:** Multivariable generalized linear models with a binary logistic distribution assessing functional dependence on assistance with basic or all activities at ICU discharge among patients discharged alive.

Variables	Adjusted OR (95% CI) for the presence of functional dependence on assistance with basic activities or all activities at ICU discharge ^a^	*P* value ^b^
Clinical Frailty Scale levels		
1 - Very fit	ref	-
2 - Well	1.143 (0.725–1.803)	0.565
3 - Managing well	2.023 (1.359–3.012)	< 0.001
4 - Vulnerable	2.774 (1.848–4.162)	< 0.001
5 - Mildly frail	4.204 (2.75–6.426)	< 0.001
6 - Moderately frail	11.052 (7.188–16.993)	< 0.001
7 - Severely frail	59.035 (35.249–98.872)	< 0.001
8 - Very severely frail	94.977 (31.591–285.539)	< 0.001
9 - Terminally ill	-	-
Sex		
Male	ref	-
Female	1.151 (1.004–1.319)	0.043
Age	1.017 (1.012–1.022)	< 0.001
Number of comorbidities		
None	ref	-
One	1.099 (0.883–1.367)	0.398
Two	1.08 (0.864–1.349)	0.501
Three or more	0.914 (0.724–1.153)	0.447
Type of hospitalization		
Elective surgical	ref	-
Emergency surgical	1.061 (0.823–1.368)	0.648
Medical	0.996 (0.853–1.163)	0.957
LLST on admission		
None	ref	-
Some	3.042 (2.080–4.451)	< 0.001
SOFA score in the first 24 h	1.128 (1.102–1.156)	< 0.001
Number of cases in the model	7114	
Bayesian information criterion (BIC)	5394.817	

^a^ Odds ratio (95% confidence interval) from the multivariable generalized linear model with a binary logistic distribution of the dependent variable, using hybrid parameter estimation and fixed-value scale, for predicting functional dependence on assistance with basic activities or all activities at ICU discharge among patients discharged alive.

^b^ Significance level of the Wald test.

^c^ Bayesian information criterion (BIC) of the multivariate generalized linear model that represents the quality of the model given its explanatory potential; the lower the BIC, the more the model is able to explain the dependent variable.

Abbreviations: CI, confidence interval; ICU, intensive care unit; LLST, limitation of life-sustaining treatment; ref, reference; SOFA, Sequential Organ Failure Assessment.

After adjustment, the relationship between the presence of one or more comorbidities and the three outcomes (transition to a higher level of functional dependence, ICU mortality, and functional dependence on assistance with basic or all activities at ICU discharge) was no longer significant (Tables 2 and 3, and 4).

In sensitivity analyses of the adjusted models considering CFS scores categorized into three levels (no frailty [scores 1–4], mild/moderate frailty [scores 5–6], and severe frailty [scores 7–9]), patients admitted with mild/moderate frailty or severe frailty, compared with those with no frailty, had significantly greater odds of death or a worse clinical condition upon ICU discharge, as measured by the adopted five-point ordinal scale (OR 2.758, 95% CI 2.455–3.098, *p* < 0.001 and OR 7.546, 95% CI 6.329–8.996, *p* < 0.001, respectively), a higher instantaneous risk of death in the ICU (HR 1.334, 95% CI 1.140–1.561, *p* < 0.001 and HR 1.395, 95% CI 1.158–1.680, *p* < 0.001, respectively), and being discharged with functional dependence on assistance with basic activities or all activities (OR 3.081, 95% CI 2.634–3.605, *p* < 0.001 and OR 30.560, 95% CI 21.768–42.904, *p* < 0.001, respectively) (Table S3, Table S4 – Figure S2 and Table S5).

The presence of some level of frailty (CFS scores 5–9) at ICU admission was significantly associated with a greater hazard of a worse clinical condition upon ICU discharge or of death (Table S6) and the odds of being discharged dependent on assistance with basic activities or all activities (Table S7, Figure S3) and increased the instantaneous risk of death in the ICU (Table S8).

## Discussion

The findings of the present study demonstrate that, among patients admitted to the ICU, the higher the previous frailty status (i.e., higher CFS score), the greater the risk of death or worse clinical condition at ICU discharge (i.e., high level of functional dependence) regardless of age, sex, number of comorbidities, type of hospitalization, limitation of life-sustaining treatment at ICU admission, and SOFA score in the first 24 h. These results corroborate those from previous studies suggesting that frailty significantly increases the risk of mortality in critically ill patients, especially among those admitted to an ICU^2,16^. Salminen et al. and Rockwood et al. demonstrated that patients with frailty, regardless of the presence of comorbidities and age, have a higher risk of death and unfavorable functional outcomes^17,18^.

The findings of the present study reinforce the growing body of evidence that frailty, as assessed by the Clinical Frailty Scale (CFS), is a risk factor for mortality and functional dependence in patients admitted to the ICU. We observed a mortality rate of 11.5% in our sample; this rate is consistent with rates reported in the literature, which range from 10% to 20% in general ICUs, especially among patients considered frail^10,11^. Indeed, the literature suggests that individuals with high CFS scores have a worse prognosis due to factors such as sarcopenia and systemic inflammation, which favor the occurrence of serious complications and increase the risk of death^12^. Frailty is strongly linked to adverse functional outcomes, impacting patients’ quality of life and independence in the long term^4,19^. The findings of the present study show an association of CFS scores with greater functional dependence at ICU discharge, with more than 40% of the patients discharged alive presenting some dependence. Regarding the clinical profile of the patients in the present study, the most common causes of ICU admission were cardiovascular diseases, followed by respiratory and neurological problems, reflecting a trend described in the literature. Notably, studies such as the one by Zampieri et al. confirm the high prevalence of cardiovascular comorbidities in critically ill patients^12,20,21^.

Another important consideration is that patients with higher CFS scores are characterized by increased physiological vulnerability due to factors such as sarcopenia and systemic inflammation, making them prone to serious complications and multiple organ failure. However, in their systematic review, Muscedere et al.^13^ found that frail and non-frail patients received similar rates of mechanical ventilation and vasopressor support, suggesting that, in the studies analyzed, perceived frailty did not necessarily lead to care limitation for these vulnerable individuals.

In all adjusted models in the present study, the CFS score emerged as an independent risk factor for level of functional dependence and mortality in the ICU, as was the SOFA score in these same models, highlighting the importance of complementing and integrating functional and social aspects with physiological ones and enhancing prognostic screening capability^22,23^. This approach is aligned with the ones recommended in recent studies, which point to including the CFS as an effective strategy to personalize and improve intensive care. The CFS can potentially optimize risk stratification, resource allocation, and post-discharge care planning, as suggested by Muscedere et al.^13^ and Bagshaw et al.^23^, who identify frailty as a key factor for critical outcomes and prolonged recovery in the ICU.

Another important consideration of the present study is the impact of frailty on the patients discharged alive from the ICU. The study revealed greater odds of discharge with significant functional dependence, especially in patients with severe frailty (for dependence on assistance with basic activities or all activities). A study by Ferrante et al.^24^ demonstrated that premorbid disability significantly influences functional trajectories and increases mortality risk among older persons after critical illness, underscoring the substantial functional decline experienced post-ICU. While their findings highlighted the poor outcomes in those with high levels of premorbid disability, the authors suggested that aggressive rehabilitation strategies, particularly for patients who worsen from minimal or mild to moderate disability to severe disability, should be investigated in future research as a potential approach to improve outcomes.

The present study has some limitations, such as the reliance on a database filled out by doctors in each health service, which is susceptible to missing information or errors in data entry. However, cases with missing essential information were excluded as described in the Sampling flowchart, and there was no missing data in the variables analyzed. We also recognize the potential risk of data-driven findings given the large number of analyses; however, we sought to mitigate this risk through a priori variable selection, predefined primary outcomes, and consistent sensitivity analyses. Although the study used data from five hospitals with different characteristics and located in a large urban center in Brazil, the findings may not be generalizable to populations in other settings due to the potential influence of healthcare diversity on the clinical outcomes of critically ill patients, which has not been addressed in this study. Future studies could overcome these limitations through multicenter analyses and longitudinal assessments of frailty.

## Conclusion

Our results confirmed the association between greater frailty at admission and worse clinical outcomes in the ICU, underscoring the utility of the CFS scoring system as an important complement to traditional severity scores. We acknowledge that the association between greater frailty and unfavorable clinical outcomes is already established in the literature. However, frailty assessment offers a more individualized understanding of the patient’s vulnerability. Thus, the implementation of frailty assessment in the ICU routine can optimize the identification of high-risk patients and inform more targeted treatment approaches.

Therefore, we suggest that frailty assessment be routinely incorporated into ICU clinical practice, assisting in the early identification of high-risk patients and potentially guiding more personalized interventions to improve their outcomes.

## Supplementary Information

Below is the link to the electronic supplementary material.

Supplementary Material 1

## Data Availability

The datasets generated and/or analysed during the current study are available in the Open Science Framework repository, https://doi.org/10.17605/OSF.IO/7KJH8.
