# Supplementary material for: Evaluation of the clinical frailty scale for predicting mortality or functional dependence at ICU discharge: A cohort study
Source: Sci Rep. 2025 Nov 12;15:39706. doi: 10.1038/s41598-025-23243-0 (PMC12612270; doi:10.1038/s41598-025-23243-0)
Supplement: Supplementary file 1 — Supplementary Material 1 [file 41598_2025_23243_MOESM1_ESM.pdf]

# Evaluation of the Clinical Frailty Scale for Predicting Mortality or Functional Dependence at ICU Discharge: A Cohort Study

Amanda Christina Kozesinski-Nakatani[1,2,3,*,+], Rafaella Stradiotto Bernardelli[1,2,+], Jeane Cristina Fonseca Vieira[2], Marcelo José Martins-Junior[2], Maria Nesryn Tiba[1], Verônica Silva Barros[2], Taher Tiba[1], Beatriz Lottermann Konzen[1], Kamila Janiscki[1], Álvaro Réa-Neto[2,+], Auristela Duarte de Lima Moser[1,+]

[1] Pontifical Catholic University of Paraná (PUCPR), Curitiba, PR, Brazil

[2] Center for Studies and Research in Intensive Care (CEPETI), Curitiba, PR, Brazil

[3] Hospital Santa Casa de Curitiba, Curitiba PR, Brazil

* akozesinski@gmail.com

+ These authors contributed equally to this work

**SUMMARY**

**Table S1** - Characteristics of the study sample and comparison of the patients' characteristics at hospitalization and outcomes, with patients categorized according to the nine levels of the Clinical Frailty Scale .................................... 3

**Table S2** – Univariable generalized linear models assessing the likelihood of death or transition to a higher level of functional dependence upon ICU discharge ............................................................................................ 5

**Table S2** – Univariable generalized linear models assessing the likelihood of death or transition to a higher level of functional dependence upon ICU discharge ............................................................................................ 6

**Table S3** – Multivariable model assessing the likelihood of death or transition to a higher level of functional dependence upon ICU discharge, considering the Clinical Frailty Scale grouped into three levels **.................................................. 7**

**Table S4** – Multivariable generalized linear model with a binary logistic distribution assessing functional dependence on assistance with basic or all activities at ICU discharge among patients discharged alive, considering the Clinical Frailty Scale grouped into three levels .................................................. 8

**Table S5** – Multivariable Cox regression model assessing the instantaneous risk of death in the ICU, considering the Clinical Frailty Scale grouped into three levels ................................................................................................ 9

**Table S6** – Multivariable generalized linear model assessing frailty as a predictor of a greater likelihood of death or transition to a higher level of functional dependence upon ICU discharge, considering the Clinical Frailty Scale dichotomized for the presence of frailty (scores 5–9) ............................ 10

**Table S7** – Multivariable generalized linear model assessing frailty as a predictor of functional dependence on assistance with basic or all activities at ICU discharge among patients discharged alive, considering the Clinical Frailty Scale (CFS) as a dichotomized variable, with frailty (scores 5–9) compared with non-frailty (scores 1–4) as the reference ......................................................... 11

**Table S8** – Multivariable Cox model assessing frailty as a predictor of a higher instantaneous risk of death in the ICU, considering the Clinical Frailty Scale as a dichotomized variable, with frailty (scores 5–9) compared with non-frailty (scores 1–4) as the reference........................................................................ 12

**Figure S1** – Comparison of the nine levels of frailty in relation to the instantaneous risk of death in the ICU............................................................ 13

**Figure S2** – Comparison of the three levels of frailty in relation to the instantaneous risk of death in the ICU............................................................ 14

**Figure S3** - Comparison of the presence versus absence of frailty at ICU admission in relation to the instantaneous risk of death in the ICU .................. 15

**Theoretical Framework for A Priori Variable Selection**...............................16

**Table S1 - Characteristics of the study sample and comparison of the patients' characteristics at hospitalization and outcomes, with patients categorized according to the nine levels of the Clinical Frailty Scale**

| Variables | Total (n=8041) | 1 - Very fit (n=559) | 2 - Well (n=1050) | 3 - Managing well (n=2695) | 4 - Vulnerable (n=1692) | 5 - Mildly frail (n=812) | 6 - Moderately frail (n=637) | 7 - Severely frail (n=417) | 8 - Very severely frail (n=144) | 9 - Terminally ill (n=35) | P value |
|---|---|---|---|---|---|---|---|---|---|---|---|
| Admission source | | | | | | | | | | | |
| Emergency room | 3787 (47.1) | 303 (54.2%) $_{a,b,c,d}$ | 538 (51.2%) $_{c,d}$ | 1108 (41.1%) $_{e}$ | 704 (41.6%) $_{e}$ | 418 (51.5%) $_{b,d}$ | 357 (56%) $_{a,b,c,d}$ | 255 (61.2%) $_{a}$ | 82 (56.9%) $_{a,b,c,d}$ | 22 (62.9%) $_{a,b,c,d,e}$ | |
| Operating room | 2591 (32.2%) | 153 (27.4%) $_{a}$ | 354 (33.7%) $_{a,b}$ | 1075 (39.9%) $_{b,c}$ | 605 (35.8%) $_{b,c}$ | 219 (27%) $_{a}$ | 124 (19.5%) $_{d}$ | 50 (12%) $_{d,e}$ | 11 (7.6%) $_{e}$ | 0 (0%) $_{d,e}$ | <0.001$^{*}$ |
| Hemodynamics service | 626 (7.8%) | 72 (12.9%) $_{a}$ | 81 (7.7%) $_{b,c,d,e}$ | 271 (10.1%) $_{a,d,e}$ | 142 (8.4%) $_{a,c,e}$ | 46 (5.7%) $_{b,c}$ | 8 (1.3%) $_{f}$ | 5 (1.2%) $_{f}$ | 1 (0.7%) $_{b,f}$ | 0 (0%) $_{a,b,c,d,e,f}$ | |
| Hospital ward | 1037 (12.9%) | 31 (5.5%) $_{a}$ | 77 (7.3%) $_{a}$ | 241 (8.9%) $_{a}$ | 241 (14.2%) $_{b}$ | 129 (15.9%) $_{b}$ | 148 (23.2%) $_{c}$ | 107 (25.7%) $_{c}$ | 50 (34.7%) $_{c}$ | 13 (37.1%) $_{c}$ | |
| SUS coverage | 3280 (40.8%) | 134 (24%) $_{a}$ | 359 (34.2%) $_{b}$ | 1110 (41.2%) $_{c}$ | 829 (49%) $_{d}$ | 397 (48.9%) $_{d,e}$ | 259 (40.7%) $_{b,c,e}$ | 139 (33.3%) $_{b,c}$ | 43 (29.9%) $_{a,b,c}$ | 10 (28.6%) $_{a,b,c,d,e}$ | <0.001$^{*}$ |
| Main reason for hospitalization | | | | | | | | | | | |
| Cardiovascular | 3099 (38.5%) | 256 (45.8%) | 426 (40.6%) | 1141 (42.3%) | 737 (43.6%) | 281 (34.6%) | 170 (26.7%) | 70 (16.8%) | 15 (10.4%) | 3 (8.6%) | |
| Respiratory | 1119 (13.9%) | 51 (9.1%) | 107 (10.2%) | 270 (10%) | 215 (12.7%) | 143 (17.6%) | 133 (20.9%) | 131 (31.4%) | 57 (39.6%) | 12 (34.3%) | |
| Neurological | 1118 (13.9%) | 75 (13.4%) | 176 (16.8%) | 462 (17.1%) | 182 (10.8%) | 98 (12.1%) | 67 (10.5%) | 49 (11.8%) | 7 (4.9%) | 2 (5.7%) | |
| Abdominal | 1108 (13.8%) | 66 (11.8%) | 142 (13.5%) | 397 (14.7%) | 240 (14.2%) | 115 (14.2%) | 88 (13.8%) | 42 (10.1%) | 12 (8.3%) | 6 (17.1%) | |
| Sepsis | 491 (6.1%) | 19 (3.4%) | 22 (2.1%) | 83 (3.1%) | 98 (5.8%) | 65 (8%) | 82 (12.9%) | 70 (16.8%) | 43 (29.9%) | 9 (25.7%) | - ‘ |
| Renal/urological | 421 (5.2%) | 24 (4.3%) | 40 (3.8%) | 139 (5.2%) | 90 (5.3%) | 45 (5.5%) | 43 (6.8%) | 36 (8.6%) | 2 (1.4%) | 2 (5.7%) | |
| Postoperative | 274 (3.4%) | 19 (3.4%) | 40 (3.8%) | 84 (3.1%) | 68 (4%) | 32 (3.9%) | 21 (3.3%) | 6 (1.4%) | 4 (2.8%) | 0 (0%) | |
| Endocrine/metabolic and electrolytic Disorder | 166 (2.1%) | 11 (2%) | 37 (3.5%) | 47 (1.7%) | 26 (1.5%) | 18 (2.2%) | 15 (2.4%) | 9 (2.2%) | 3 (2.1%) | 0 (0%) | |
| Exogenous intoxication | 102 (1.3%) | 25 (4.5%) | 36 (3.4%) | 26 (1%) | 10 (0.6%) | 2 (0.2%) | 2 (0.3%) | 1 (0.2%) | 0 (0%) | 0 (0%) | |
| Trauma | 85 (1.1%) | 8 (1.4%) | 12 (1.1%) | 21 (0.8%) | 22 (1.3%) | 9 (1.1%) | 10 (1.6%) | 3 (0.7%) | 0 (0%) | 0 (0%) | |
| Gynecological | 58 (0.7%) | 5 (0.9%) | 12 (1.1%) | 25 (0.9%) | 4 (0.2%) | 4 (0.5%) | 6 (0.9%) | 0 (0%) | 1 (0.7%) | 1 (2.9%) | |
| VAD on admission | 1505 (18.7%) | 54 (9.7%) $_{a}$ | 114 (10.9%) $_{a}$ | 438 (16.3%) $_{b}$ | 384 (22.7%) $_{c}$ | 203 (25%) $_{c}$ | 152 (23.9%) $_{c}$ | 104 (24.9%) $_{c}$ | 47 (32.6%) $_{c}$ | 9 (25.7%) $_{a,b,c}$ | <0.001$^{*}$ |
| IMV on admission | 1296 (16.1%) | 63 (11.3%) $_{a}$ | 121 (11.5%) $_{a}$ | 406 (15.1%) $_{a,b}$ | 325 (19.2%) $_{c}$ | 158 (19.5%) $_{b,c}$ | 99 (15.5%) $_{a,b,c}$ | 90 (21.6%) $_{c}$ | 30 (20.8%) $_{a,b,c}$ | 4 (11.4%) $_{a,b,c}$ | <0.001$^{*}$ |
| GCS on admission | 15 (14–15) | 15 (15–15) $_{a}$ | 15 (15–15) $_{a,b}$ | 15 (15–15) $_{a,b,c}$ | 15 (14–15) $_{c}$ | 15 (14–15) $_{e}$ | 14 (13–15) $_{f}$ | 13 (10–14) $_{g}$ | 11 (8–14) $_{h}$ | 10 (8–14) $_{h}$ | <0.001$^{\#}$ |
| Mean SOFA score at hospitalization | 1.5 (0.3–3.8) | 0.5 (0–1.8) $_{a}$ | 0.8 (0–2) $_{a}$ | 1 (0–2.9) $_{c}$ | 2 (0.7–4) $_{d}$ | 2.5 (1–5) $_{e}$ | 3 (1.5–5.1) $_{e}$ | 3.9 (2.2–6.3) $_{g}$ | 5.4 (3.6–9) $_{h}$ | 6 (4–9) $_{g,h}$ | <0.001$^{\#}$ |
| Median SOFA score at hospitalization | 1.5 (0–4) | 0.5 (0–2) $_{a}$ | 1 (0–2) $_{a}$ | 1 (0–3) $_{c}$ | 2 (0.5–4) $_{d}$ | 2.5 (1–5) $_{e}$ | 3 (1–5) $_{e}$ | 4 (2–6) $_{g}$ | 5.3 (3–9) $_{h}$ | 6 (4–9) $_{g,h}$ | <0.001$^{\#}$ |
| Maximum SOFA score at hospitalization | 2 (1–5) | 1 (0–3) $_{a}$ | 1 (0–3) $_{a}$ | 1 (0–4) $_{c}$ | 3 (1–6) $_{d}$ | 4 (2–8) $_{e}$ | 4 (2–8) $_{e}$ | 5 (3–9) $_{g}$ | 7 (5–11) $_{h}$ | 7 (5–11) $_{g,h}$ | <0.001$^{\#}$ |
| SOFA score in the last 24 hours of hospitalization | 1 (0–3) | 0 (0–1) $_{a}$ | 0 (0–1) $_{a}$ | 1 (0–2) | 1 (0–3) | 2 (1–4) $_{e}$ | 2 (1–5) $_{e}$ | 3 (2–6) $_{g}$ | 5 (3–10) $_{h}$ | 7 (4–11) $_{h}$ | <0.001$^{\#}$ |
| Use of IUC | 1868 (23.2%) | 68 (12.2%) $_{a}$ | 178 (17%) $_{a,b}$ | 478 (17.7%) $_{b}$ | 420 (24.8%) $_{c}$ | 263 (32.4%) $_{d}$ | 243 (38.1%) $_{d}$ | 162 (38.8%) $_{d}$ | 45 (31.3%) $_{c,d}$ | 11 (31.4%) $_{b,c,d}$ | <0.001$^{*}$ |
| Use of NET | 767 (9.5%) | 33 (5.9%) $_{a,b}$ | 69 (6.6%) $_{b,c}$ | 145 (5.4%) $_{b}$ | 175 (10.3%) $_{a,d}$ | 106 (13.1%) $_{d,e}$ | 113 (17.7%) $_{e,f}$ | 98 (23.5%) $_{f}$ | 21 (14.6%) $_{d,e,f}$ | 7 (20%) $_{c,d,e,f}$ | <0.001$^{*}$ |
| Use of CVA | 1450 (18%) | 47 (8.4%) $_{a}$ | 134 (12.8%) $_{a,b}$ | 366 (13.6%) $_{b}$ | 346 (20.4%) $_{c}$ | 207 (25.5%) $_{c,d}$ | 180 (28.3%) $_{d}$ | 119 (28.5%) $_{d}$ | 43 (29.9%) $_{c,d}$ | 8 (22.9%) $_{a,b,c,d}$ | <0.001$^{*}$ |
| Use of IAPM | 1362 (16.9%) | 42 (7.5%) $_{a}$ | 127 (12.1%) $_{a,b}$ | 368 (13.7%) $_{b}$ | 336 (19.9%) $_{c}$ | 192 (23.6%) $_{c,d}$ | 166 (26.1%) $_{d}$ | 98 (23.5%) $_{c,d}$ | 30 (20.8%) $_{b,c,d}$ | 3 (8.6%) $_{a,b,c,d}$ | <0.001$^{*}$ |

| | | | | | | | | | | | |
|---|---|---|---|---|---|---|---|---|---|---|---|
| Use of RRT | 243 (3%) | 10 (1.8%) $_{a,b,c,d}$ | 21 (2%) $_{c,d}$ | 61 (2.3%) $_{b,d}$ | 74 (4.4%) $_{a}$ | 34 (4.2%) $_{a,b,c,d}$ | 24 (3.8%) $_{a,b,c,d}$ | 14 (3.4%) $_{a,b,c,d}$ | 4 (2.8%) $_{a,b,c,d}$ | 1 (2.9%) $_{a,b,c,d}$ | <0.001* |
| Use of MV | 1233 (15.3%) | 38 (6.8%) $_{a}$ | 121 (11.5%) $_{a,b}$ | 318 (11.8%) $_{b}$ | 291 (17.2%) $_{c}$ | 172 (21.2%) $_{c,d}$ | 147 (23.1%) $_{d}$ | 108 (25.9%) $_{d}$ | 37 (25.7%) $_{c,d}$ | 1 (2.9%) $_{a,b,c,d}$ | <0.001* |
| Nosocomial infection | 1467 (18.2%) | 76 (13.6%) $_{a,b}$ | 121 (11.5%) $_{b}$ | 311 (11.5%) $_{b}$ | 311 (18.4%) $_{a,c}$ | 190 (23.4%) $_{c}$ | 206 (32.3%) $_{d}$ | 176 (42.2%) $_{e}$ | 63 (43.8%) $_{d,e}$ | 13 (37.1%) $_{c,d,e}$ | <0.001* |
| Had CRA | 941 (11.7%) | 20 (3.6%) $_{a}$ | 52 (5%) $_{a}$ | 156 (5.8%) $_{a}$ | 214 (12.6%) $_{b}$ | 143 (17.6%) $_{c}$ | 142 (22.3%) $_{c,d}$ | 116 (27.8%) $_{d}$ | 76 (52.8%) $_{e}$ | 22 (62.9%) $_{e}$ | <0.001* |
| LLST at ICU discharge or death | | | | | | | | | | | |
| A | 7048 (87.7%) | 542 (97%) $_{a}$ | 1020 (97.1%) $_{a}$ | 2579 (95.7%) $_{a}$ | 1508 (89.1%) $_{b}$ | 672 (82.8%) $_{c}$ | 460 (72.2%) $_{d}$ | 231 (55.4%) $_{e}$ | 34 (23.6%) $_{f}$ | 2 (5.7%) $_{f}$ | |
| B | 536 (6.7%) | 10 (1.8%) $_{a}$ | 19 (1.8%) $_{a}$ | 65 (2.4%) $_{a}$ | 102 (6%) $_{b}$ | 71 (8.7%) $_{b}$ | 106 (16.6%) $_{c}$ | 105 (25.2%) $_{d}$ | 53 (36.8%) $_{d}$ | 5 (14.3%) $_{b,c,d}$ | <0.001* |
| C/D | 757 (5.7%) | 7 (1.3%) $_{a}$ | 11 (11%) $_{a}$ | 51 (1.9%) $_{a}$ | 82 (4.8%) $_{b}$ | 69 (8.5%) $_{c}$ | 71 (11.1%) $_{c}$ | 81 (19.4%) $_{d}$ | 57 (39.6%) $_{e}$ | 28 (80%) $_{f}$ | |
| GCS among the 7114 discharged patients | 15 (15–15) | 15 (15–15) | 15 (15–15) | 15 (15–15) | 15 (15–15) | 15 (15–15) | 15 (14–15) | 14 (12–15) | 12 (10–14) | 10 (8–11) | <0.001# |
| LLST among patients discharged alive from the ICU (n = 7114) | | | | | | | | | | | |
| A | 6753 (94.9%) | 537 (99.4%) | 991 (99.1%) | 2518 (99%) | 1432 (96.7%) | 622 (92.8%) | 426 (85.9%) | 203 (67.4%) | 23 (33.3%) | 1 (7.7%) | -” |
| B | 244 (3.4%) | 2 (0.4%) | 6 (0.6%) | 19 (0.7%) | 31 (2.1%) | 30 (4.5%) | 53 (10.7%) | 69 (22.9%) | 30 (43.5%) | 4 (30.8%) | |
| C/D | 117 (1.7%) | 1 (0.2%) | 3 (0.3%) | 7 (0.3%) | 18 (1.2%) | 18 (2.7%) | 17 (3.4%) | 29 (9.6%) | 16 (23.2%) | 8 (61.5%) | |
| LLST among patients who died in the ICU (n = 927) | | | | | | | | | | | |
| A | 295 (31.8%) | 5 (26.3%) $_{a,b,c,d,e,f}$ | 29 (58%) $_{f}$ | 61 (40.4%) $_{e,f}$ | 76 (36%) $_{c,d,e,f}$ | 50 (35.2%) $_{b,d,e,f}$ | 34 (24.1%) $_{a,b,c,d,e}$ | 28 (24.1%) $_{a,b,c,d,e}$ | 11 (14.7%) $_{a}$ | 1 (4.5%) $_{a,b,c,d}$ | <0.001 * |
| B | 292 (31.5%) | 8 (42.1%) $_{a}$ | 13 (26%) $_{a}$ | 46 (30.5%) $_{a}$ | 71 (33.6%) $_{a}$ | 41 (28.9%) $_{a}$ | 53 (37.6%) $_{a}$ | 36 (31%) $_{a}$ | 23 (30.7%) $_{a}$ | 1 (4.5%) $_{a}$ | |
| C/D | 340 (36.7%) | 6 (31.6%) $_{a,b,c}$ | 8 (16%) $_{c}$ | 44 (29.1%) $_{b,c}$ | 64 (30.3%) $_{b,c}$ | 51 (35.9%) $_{a,b,c}$ | 54 (38.3%) $_{a,b,c}$ | 52 (44.8%) $_{a,b}$ | 41 (54.7%) $_{a,d}$ | 20 (90.9%) $_{d}$ | |

Age is reported as mean ± standard deviation, while the other quantitative variables are reported as mean or median (interquartile range). Categorical variables are described as frequency (percentage). The categories of LLST included the following: A, all necessary measures; B, all necessary measures but not including cardiopulmonary resuscitation; C, withholding of support; D, withdrawal of support; E, brain death.

# Statistical significance determined by the nonparametric Kruskal-Wallis test followed by *post hoc* analysis using Dunn’s test with a Bonferroni-adjusted significance level.

* Statistical significance determined by the chi-square test followed by a row-by-row comparison of case proportions using the Z test, with the significance value adjusted by the Bonferroni method.

-” The chi-square test could not be applied due to some cells with an expected frequency lower than 1.

Subscript letters: The results of two-by-two comparisons (post hoc) are expressed in letters, in which equal letters indicate that there is no significant difference between the groups and different letters indicate that there is significant difference (p<0.05).

Abbreviations: APACHE II, Acute Physiology and Chronic Health Evaluation II; CRA, cardiorespiratory arrest; CVA, central venous access; ECG, electrocardiogram; ICU, intensive care unit; IUC, indwelling urinary catheter; IMV, invasive mechanical ventilation; LLST, limitation of life-sustaining treatment; IAPM, invasive arterial pressure monitoring; MV, mechanical ventilation; NET, nasoenteric tube; SOFA, Sequential Organ Failure Assessment; SUS, Brazilian Unified Health System; VAD, vasoactive drug.

**Table S2 – Univariable generalized linear models assessing the likelihood of death or transition to a higher level of functional dependence upon ICU discharge**

| Variables | Number of cases (in each row) | 1 - Fully independente [a] | 2 - Dependent on assistance with complex activities [a] | 3 - Dependent on assistance with basic activities [a] | 4 - Dependent on assistance with all activities [a] | 5 – Death [a] | Unadjusted OR (95% CI) assessing the likelihood of death or transition to a higher level of functional dependence at ICU discharge [b] | P value [c] |
|---|---|---|---|---|---|---|---|---|
| Clinical Frailty Scale levels | | | | | | | | |
| 1 - Very fit | 559 | 400 (71.6%) | 111 (19.9%) | 21 (3.8%) | 8 (1.4%) | 19 (3.4%) | ref | |
| 2 - Well | 1050 | 614 (58.5%) | 322 (30.7%) | 57 (5.4%) | 7 (0.7%) | 50 (4.8%) | 1.229 (0.724–2.085) | 0.446 |
| 3 - Managing well | 2695 | 1176 (43.6%) | 1053 (39.1%) | 287 (10.6%) | 28 (1%) | 151 (5.6%) | 1.536 (0.953–2.475) | 0.078 |
| 4 - Vulnerable | 1692 | 503 (29.7%) | 712 (42.1%) | 225 (13.3%) | 41 (2.4%) | 211 (12.5%) | 2.502 (1.563–4.003) | <0.001 |
| 5 - Mildly frail | 812 | 147 (18.1%) | 334 (41.1%) | 134 (16.5%) | 55 (6.8%) | 142 (17.5%) | 2.891 (1.79–4.67) | <0.001 |
| 6 - Moderately frail | 637 | 52 (8.2%) | 180 (28.3%) | 183 (28.7%) | 81 (12.7%) | 141 (22.1%) | 3.549 (2.197–5.734) | <0.001 |
| 7 - Severely frail | 417 | 10 (2.4%) | 30 (7.2%) | 117 (28.1%) | 144 (34.5%) | 116 (27.8%) | 3.457 (2.125–5.623) | <0.001 |
| 8 - Very severely frail | 144 | 1 (0.7%) | 3 (2.1%) | 8 (5.6%) | 57 (39.6%) | 75 (52.1%) | 7.678 (4.636–12.715) | <0.001 |
| 9 - Terminally ill | 35 | 0 (0%) | 0 (0%) | 1 (2.9%) | 12 (34.3%) | 22 (62.9%) | 12.176 (6.584–22.516) | <0.001 |
| Categorized CFS | | | | | | | | |
| No frailty (scores 1–4) | 5996 | 2693 (44.9%) | 2198 (36.7%) | 590 (9.8%) | 84 (1.4%) | 431 (7.2%) | ref | |
| Mild/moderate frailty (scores 5–6) | 1449 | 199 (13.7%) | 514 (35.5%) | 317 (21.9%) | 136 (9.4%) | 283 (19.5%) | 4.688 (4.206–5.226) | <0.001 |
| Severe frailty (scores 7–9) | 596 | 11 (1.8%) | 33 (5.5%) | 126 (21.1%) | 213 (35.7%) | 213 (35.7%) | 18.235 (15.611–21.301) | <0.001 |
| Presence of frailty (CFS scores 5–9) | | | | | | | | |
| No | 5996 | 2693 (44.9%) | 2198 (36.7%) | 590 (9.8%) | 84 (1.4%) | 431 (7.2%) | ref | |
| Yes | 2045 | 210 (10.3%) | 547 (26.7%) | 443 (21.7%) | 349 (17.1%) | 496 (24.3%) | 1.323 (1.221–1.432) | <0.001 |
| Age | 8041 | 2903 (59.2 ± 16.7) | 2745 (66.9 ± 16.4) | 1033 (72.5 ± 16.5) | 433 (74.8 ± 17) | 927 (72.9 ± 14.3) | 1.037 (1.034–1.040) | <0.001 |

**Table S2 – Univariable generalized linear models assessing the likelihood of death or transition to a higher level of functional dependence upon ICU discharge**

| | | | | | | | | |
|---|---|---|---|---|---|---|---|---|
| Sex | | | | | | | | |
| Male | 3910 | 1577 (40.3%) | 1261 (32.3%) | 458 (11.7%) | 173 (4.4%) | 441 (11.3%) | ref | |
| Female | 4131 | 1326 (32.1%) | 1484 (35.9%) | 575 (13.9%) | 260 (6.3%) | 486 (11.8%) | 1.323 (1.221–1.432) | <0.001 |
| Number of comorbidities | | | | | | | | |
| None | 1487 | 743 (50%) | 514 (34.6%) | 125 (8.4%) | 33 (2.2%) | 72 (4.8%) | ref | |
| One | 2415 | 930 (38.5%) | 832 (34.5%) | 302 (12.5%) | 113 (4.7%) | 238 (9.9%) | 2.981 (2.629–3.38) | <0.001 |
| Two | 2154 | 672 (31.2%) | 767 (35.6%) | 304 (14.1%) | 139 (6.5%) | 272 (12.6%) | 2.319 (2.052–2.621) | <0.001 |
| Three or more | 1985 | 558 (28.1%) | 632 (31.8%) | 302 (15.2%) | 148 (7.5%) | 345 (17.4%) | 1.701 (1.509–1.918) | <0.001 |
| Type of ICU admission | | | | | | | | |
| Elective surgical | 2423 | 932 (38.5%) | 1047 (43.2%) | 300 (12.4%) | 59 (2.4%) | 85 (3.5%) | ref | |
| Emergency surgical | 713 | 204 (28.6%) | 276 (38.7%) | 81 (11.4%) | 40 (5.6%) | 112 (15.7%) | 1.794 (1.544 - 2.083) | <0.001 |
| Medical | 4905 | 1767 (36%) | 1422 (29%) | 652 (13.3%) | 334 (6.8%) | 730 (14.9%) | 1.578 (1.446 - 1.723) | <0.001 |
| LLST at admission | | | | | | | | |
| None | 7670 | 2887 (37.6%) | 2705 (35.3%) | 956 (12.5%) | 340 (4.4%) | 782 (10.2%) | ref | |
| Some | 371 | 16 (8.8%) | 40 (22.8%) | 77 (31.2%) | 93 (47.3%) | 145 (89.7%) | 8.576 (7.127 - 10.32) | <0.001 |
| SOFA score in the first 24 hours; n (median [IQR]) | 8041 | 2903 (1 [0–2]) | 2745 (1 [0–4]) | 1033 (2 [1–4]) | 433 (4 [2–6]) | 927 (7 [4–10]) | 1.345 (1.326–1.364) | <0.001 |

[a] Values are shown as number (percentage), number (mean ± standard deviation), or number (median value [interquartile range]).

[b] Odds ratio (95% confidence interval) from the univariable analysis of a generalized linear model with ordinal logistic regression for the dependent variable, using hybrid parameter estimation and a fixed-value scale, assessing the association with the likelihood of a higher level of functional dependence and mortality at ICU discharge.

[c] Significance level of the Wald test.

Abbreviations: CFS, Clinical Frailty Scale; CI, confidence interval; ICU, intensive care unit; IQR, interquartile range; LLST, limitation of life-sustaining treatment; n, number; OR, odds ratio; ref, reference; SOFA, Sequential Organ Failure Assessment.

**Table S3 – Multivariable model assessing the likelihood of death or transition to a higher level of functional dependence upon ICU discharge, considering the Clinical Frailty Scale grouped into three levels**

| Variables | Adjusted OR (95% CI) for death or transition to a higher level of functional dependence upon ICU discharge [a] | P value [b] |
|---|---|---|
| Categorized CFS | | |
| No frailty (scores 1–4) | ref | |
| Mild/moderate frailty (scores 5–6) | 2.758 (2.455–3.098) | <0.001 |
| Severe frailty (scores 7–9) | 7.546 (6.329–8.996) | <0.001 |
| Sex | | |
| Male | ref | |
| Female | 1.297 (1.193–1.411) | <0.001 |
| Age | 1.021 (1.018–1.024) | <0.001 |
| Number of comorbidities | | |
| None | ref | |
| One | 1.131 (0.996–1.285) | 0.057 |
| Two | 1.118 (0.98–1.277) | 0.098 |
| Three or more | 1.001 (0.871–1.15) | 0.992 |
| Type of hospitalization | | |
| Elective surgical | ref | |
| Emergency surgical | 1.317 (1.124–1.542) | <0.001 |
| Clinical | 0.986 (0.896–1.085) | 0.779 |
| LLST on admission | | |
| None | ref | |
| Some | 2.019 (1.64–2.484) | <0.001 |
| SOFA score in the first 24 hours | 1.288 (1.268–1.307) | <0.001 |
| Number of cases in the model | 8041 | |
| Bayesian information criterion (BIC) | 17077.019 | |

[a] Odds ratio (95% confidence interval) from the multivariable generalized linear model with ordinal logistic regression, using hybrid parameter estimation and a fixed-value scale, assessing the likelihood of death or transition to a higher level of functional dependence upon ICU discharge.

[b] Significance level of the Wald test.

[c] Bayesian information criterion (BIC) of the multivariable generalized linear model, representing the model's quality based on its explanatory potential; the lower the BIC, the better the model's ability to explain the dependent variable.

Abbreviations: CFS, Clinical Frailty Scale; CI, confidence interval; ICU, intensive care unit; LLST, limitation of life-sustaining treatment; ref, reference; SOFA, Sequential Organ Failure Assessment.

**Table S4 – Multivariable generalized linear model with a binary logistic distribution assessing functional dependence on assistance with basic or all activities at ICU discharge among patients discharged alive, considering the Clinical Frailty Scale grouped into three levels**

| Variables | Adjusted OR (95% CI) for the presence of functional dependence on assistance with basic or all activities at ICU discharge [a] | P value [b] |
|---|---|---|
| Categorized CFS | | |
| No frailty (scores 1–4) | | |
| Mild/moderate frailty (scores 5–6) | 3.081 (2.634–3.605) | <0.001 |
| Severe frailty (scores 7–9) | 30.56 (21.768–42.904) | <0.001 |
| Sex | | |
| Male | ref | |
| Female | 1.153 (1.008–1.32) | 0.038 |
| Age | 1.021 (1.016–1.026) | <0.001 |
| Number of comorbidities | | |
| None | ref | |
| One | 1.194 (0.963–1.48) | 0.106 |
| Two | 1.154 (0.926–1.438) | 0.203 |
| Three or more | 1.003 (0.798–1.262) | 0.977 |
| Type of hospitalization | | |
| Elective surgical | ref | |
| Emergency surgical | 1.054 (0.819–1.356) | 0.682 |
| Medical | 0.984 (0.845–1.147) | 0.838 |
| LLST on admission | | |
| None | ref | |
| Some | 3.291 (2.271–4.77) | <0.001 |
| SOFA score in the first 24 hours | 1.136 (1.109–1.163) | <0.001 |
| Number of cases in the model | 7114 | |
| Bayesian information criterion (BIC) | 5100.754 | |

[a] Odds ratio (95% confidence interval) from the multivariable generalized linear models with binary logistic regression, using hybrid parameter estimation and a fixed-value scale, assessing the presence of functional dependence on assistance with basic or all activities at ICU discharge among patients discharged alive.
[b] Significance level of the Wald test.
[c] Bayesian information criterion (BIC) of the multivariable generalized linear model, representing the model's quality based on its explanatory potential; the lower the BIC, the better the model's ability to explain the dependent variable.
Abbreviations: CFS, Clinical Frailty Scale; CI, confidence interval; ICU, intensive care unit; LLST, limitation of life-sustaining treatment; ref, reference; SOFA, Sequential Organ Failure Assessment.

**Table S5 – Multivariable Cox regression model assessing the instantaneous risk of death in the ICU, considering the Clinical Frailty Scale grouped into three levels**

| Variables | Adjusted HR (95% CI) for ICU mortality [a] | P value [b] |
|---|---|---|
| Categorized CFS | | |
| No frailty (scores 1–4) | ref | |
| Mild/moderate frailty (scores 5–6) | 1.334 (1.140–1.561) | <0.001 |
| Severe frailty (scores 7–9) | 1.395 (1.158–1.680) | <0.001 |
| Sex | | |
| Male | ref | |
| Female | 1.2 (1.053–1.368) | 0.006 |
| Age | 1.012 (1.007–1.018) | <0.001 |
| Number of comorbidities | | |
| None | ref | |
| One | 1.158 (0.887–1.514) | 0.281 |
| Two | 1.05 (0.804–1.37) | 0.721 |
| Three or more | 0.998 (0.766–1.3) | 0.989 |
| Type of hospitalization | | |
| Elective surgical | ref | |
| Emergency surgical | 1.842 (1.385–2.45) | <0.001 |
| Medical | 1.576 (1.251–1.986) | <0.001 |
| LLST on admission | | |
| None | ref | |
| Some | 2.477 (2.029–3.025) | <0.001 |
| SOFA score in the first 24 hours | 1.183 (1.165–1.202) | <0.001 |
| Number of cases in the model | 8041 | |

[a] Hazard ratio (95% confidence interval) from the multivariable Cox regression model assessing ICU mortality.

[b] Significance level of the Wald test.

Abbreviations: CFS, Clinical Frailty Scale; CI, confidence interval; HR, hazard ratio; ICU, intensive care unit; LST, limitation of life-sustaining treatment; ref, reference; SOFA, Sequential Organ Failure Assessment.

**Table S6 – Multivariable generalized linear model assessing frailty as a predictor of a greater likelihood of death or transition to a higher level of functional dependence upon ICU discharge, considering the Clinical Frailty Scale dichotomized for the presence of frailty (scores 5–9)**

| Variables | Adjusted OR (95% CI) for the likelihood of death or transition to a higher level of functional dependence upon ICU discharge [a] | P value [b] |
|---|---|---|
| Frailty | | |
| Absent (scores 1–4) | ref | |
| Present (scores 5–9) | 3.476 (3.116 - 3.877) | <0.001 |
| Sex | | |
| Male | ref | |
| Female | 1.315 (1.209 - 1.43) | <0.001 |
| Age | 1.021 (1.018 - 1.024) | <0.001 |
| Number of comorbidities | | |
| None | ref | |
| One | 1.142 (1.006 - 1.297) | 0.04 |
| Two | 1.116 (0.978 - 1.274) | 0.104 |
| Three or more | 1.015 (0.883 - 1.166) | 0.839 |
| Type of hospitalization | | |
| Elective surgical | ref | |
| Emergency surgical | 1.306 (1.115 - 1.53) | <0.001 |
| Medical | 1.012 (0.919 - 1.113) | 0.811 |
| LLST on admission | | |
| None | ref | |
| Some | 2.655 (2.174 - 3.242) | <0.001 |
| SOFA score in the first 24 hours | 1.294 (1.274 - 1.313) | <0.001 |
| Number of cases in the model | 8041 | |
| Bayesian information criterion (BIC) | 17056.702 | |

[a] Odds ratio (95% confidence interval) from the multivariable generalized linear models with ordinal logistic regression, using hybrid parameter estimation and a fixed-value scale, assessing the likelihood of death or transition to a higher level of functional dependence upon ICU discharge (1: Fully independent; 2: Dependent on assistance with complex activities; 3: Dependent on assistance with basic activities; 4: Dependent on assistance with all activities; and 5: Death).

[b] Significance level of the Wald test.

[c] Bayesian information criterion (BIC) of the multivariable generalized linear model, representing the model's quality based on its explanatory potential; the lower the BIC, the better the model's ability to explain the dependent variable.

Abbreviations: CI, confidence interval; ICU, intensive care unit; LLST, limitation of life-sustaining treatment; OR, odds ratio; ref, reference; SOFA, Sequential Organ Failure Assessment.

**Table S7 – Multivariable generalized linear model assessing frailty as a predictor of functional dependence on assistance with basic or all activities at ICU discharge among patients discharged alive, considering the Clinical Frailty Scale (CFS) as a dichotomized variable, with frailty (scores 5–9) compared with non-frailty (scores 1–4) as the reference**

| Variables | Adjusted OR (95% CI) for the presence of functional dependence on assistance with basic or all activities at ICU discharge [a] | P value [b] |
|---|---|---|
| Frailty | | |
| Absent (scores 1–4) | ref | |
| Present (scores 5–9) | 4.627 (4.006–5.343) | <0.001 |
| Sex | | |
| Male | ref | |
| Female | 1.18 (1.035–1.346) | 0.013 |
| Age | 1.019 (1.015–1.024) | <0.001 |
| Number of comorbidities | | |
| None | ref | |
| One | 1.252 (1.012–1.55) | 0.039 |
| Two | 1.180 (0.949–1.468) | 0.137 |
| Three or more | 1.053 (0.84–1.32) | 0.654 |
| Type of hospitalization | | |
| Elective surgical | ref | |
| Emergency surgical | 1.059 (0.825–1.359) | 0.654 |
| Medical | 1.066 (0.917–1.239) | 0.405 |
| LLST on admission | | |
| None | ref | |
| Some | 4.503 (3.203–6.33) | <0.001 |
| SOFA score in the first 24 hours | 1.149 (1.123–1.175) | <0.001 |
| Number of cases in the model | 7114 | |
| Bayesian information criterion (BIC) | 5268.884 | |

[a] Odds ratio (95% CI) from the multivariable generalized linear model with binary logistic regression for the dependent variable, using hybrid parameter estimation and a fixed-value scale, assessing the presence of functional dependence in basic or all activities at ICU discharge among patients discharged alive.

[b] Significance level of the Wald test.

[c] Bayesian information criterion (BIC) of the multivariable generalized linear model, representing the model's quality based on its explanatory potential; the lower the BIC, the better the model's ability to explain the dependent variable.

**Table S8 – Multivariable Cox model assessing frailty as a predictor of a higher instantaneous risk of death in the ICU, considering the Clinical Frailty Scale as a dichotomized variable, with frailty (scores 5–9) compared with non-frailty (scores 1–4) as the reference**

| Variables | Adjusted HR (95% CI) for ICU mortality [a] | P value [b] |
|---|---|---|
| Frailty | | |
| Absent (scores 1–4) | ref | |
| Present (scores 5–9) | 1.355 (1.176–1.562) | <0.001 |
| Sex | | |
| Male | ref | |
| Female | 1.202 (1.054–1.37) | 0.006 |
| Age | 1.013 (1.007–1.018) | <0.001 |
| Number of comorbidities | | |
| None | ref | |
| One | 1.159 (0.887–1.514) | 0.279 |
| Two | 1.047 (0.802–1.367) | 0.734 |
| Three or more | 0.998 (0.766–1.3) | 0.988 |
| Type of hospitalization | | |
| Elective surgical | ref | |
| Emergency surgical | 1.841 (1.384–2.449) | <0.001 |
| Medical | 1.579 (1.254–1.99) | <0.001 |
| LLST on admission | | |
| None | ref | |
| Some | 2.508 (2.069–3.041) | <0.001 |
| SOFA score in the first 24 hours | 1.183 (1.165–1.202) | <0.001 |
| Number of cases in the model | 8041 | |

[a] Hazard ratio (95% confidence interval) from the multivariable Cox regression model for ICU mortality.
[b] Significance level of the Wald test.
Abbreviations: CFS, Clinical Frailty Scale; HR, hazard ratio; ICU, intensive care unit; LLST, limitation of life-sustaining treatment; ref., reference; SOFA, Sequential Organ Failure Assessment.

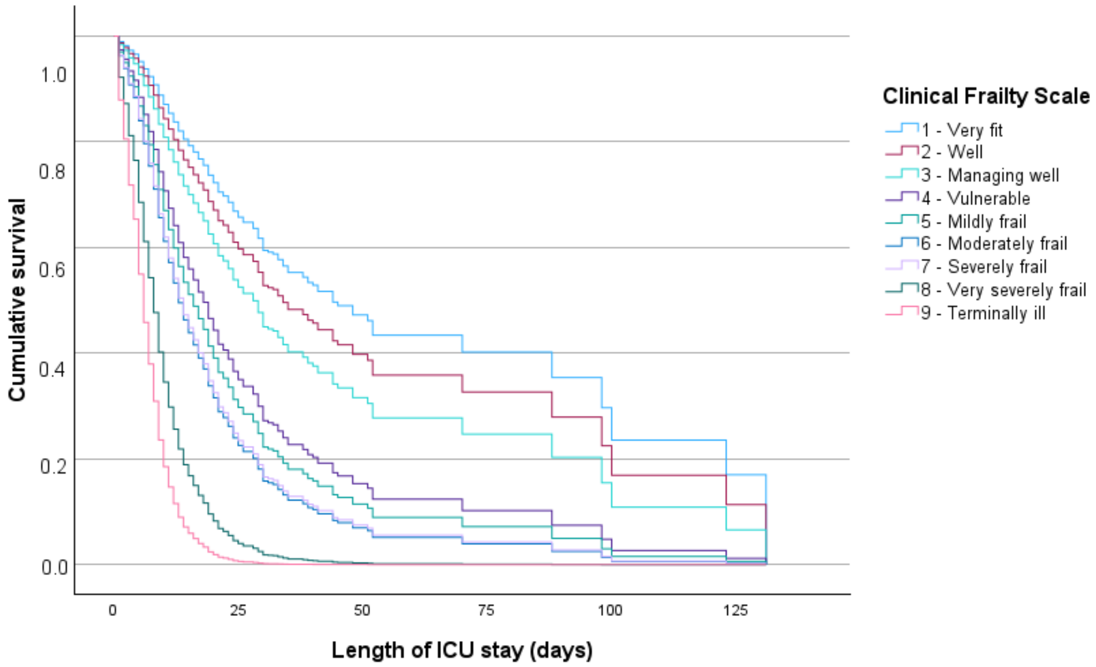


**Figure S1 – Comparison of the nine levels of frailty in relation to the instantaneous risk of death in the ICU.** Outcome presented in cumulative survival over time for each of the nine levels of frailty, based on the univariable Cox regression model for ICU mortality.

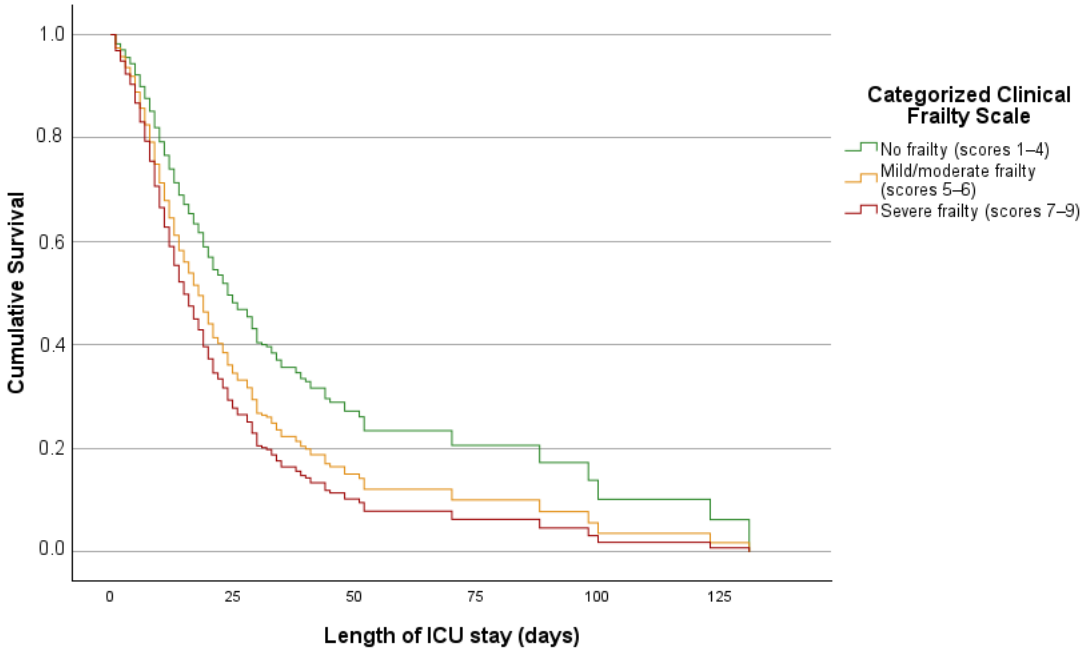


**Figure S2 – Comparison of the three levels of frailty in relation to the instantaneous risk of death in the ICU.** Cumulative survival over time is presented for each group, based on the multivariable Cox regression model for ICU mortality adjusted for sex, age, number of comorbidities, type of hospitalization, and presence of limitation of life-sustaining treatment at ICU admission.

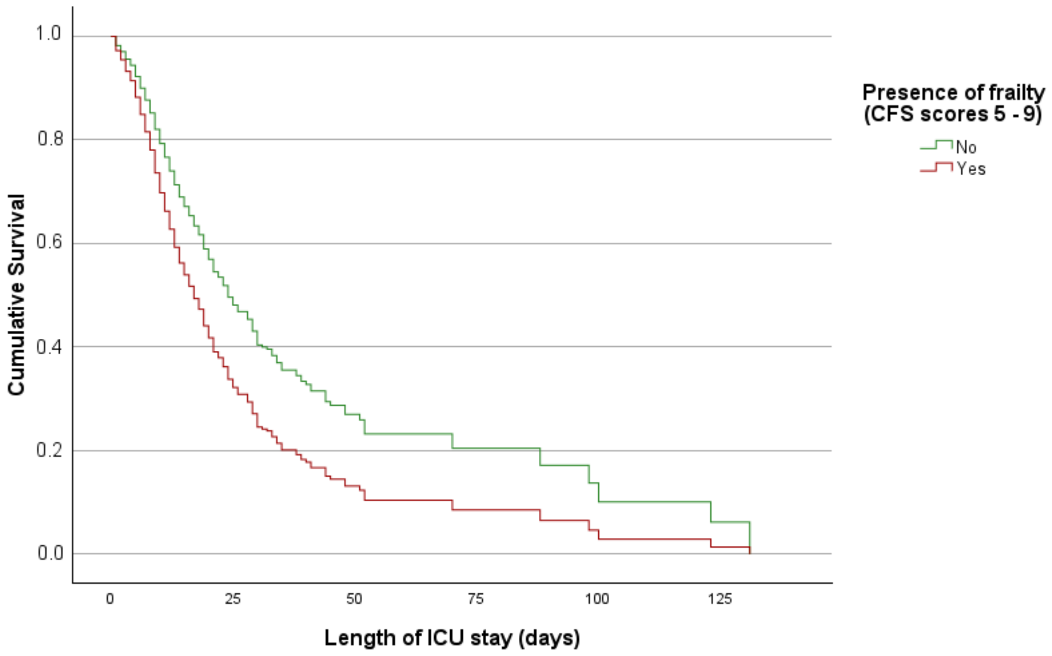


**Figure S3 - Comparison of the presence versus absence of frailty at ICU admission in relation to the instantaneous risk of death in the ICU.** Cumulative survival over time is presented for each group, based on the multivariable Cox regression model for ICU mortality adjusted for sex, age, number of comorbidities, type of hospitalization, and presence of limitation of life-sustaining treatment at ICU admission.

**Theoretical Framework for A Priori Variable Selection**

Our multivariable models incorporated adjustment variables selected a priori based on established critical care evidence, serving two key purposes: (1) systematically including factors consistently associated with ICU outcomes, and (2) controlling for potential confounding to precisely estimate frailty's independent association with outcomes via the Clinical Frailty Scale (CFS). Each variable was chosen for its demonstrated role as either confounder or effect modifier in the frailty-outcome relationship.

Building on this foundation, advanced chronological age represents one of the most robust predictors of adverse ICU outcomes. Elderly patients typically present with greater comorbidity burdens, diminished physiological reserve, and increased susceptibility to complications [13-15,22,24]. While age and frailty frequently coexist, frailty represents a distinct construct of biological vulnerability assessed through CFS evaluation of comorbidity burden, functional status, and cognitive function [6,7]. Our age adjustment enables separation of frailty's effects from age-related physiological changes, ensuring CFS-outcome associations reflect true vulnerability rather than chronological aging [7,8,18,19].

Beyond chronological factors, sex-related differences influence critical care outcomes through biological pathways (hormonal modulation of immune/cardiovascular function) and psychosocial factors [14,15]. Our sex adjustment accounts for these inherent variations while isolating frailty's independent prognostic value from sex-associated outcome disparities [13-15].

Equally important is the consideration of comorbidity burden. Each additional chronic condition (e.g., diabetes, heart failure, COPD) progressively erodes physiological reserve, increasing vulnerability to acute stressors [13,14,23]. While frailty often coexists with polymorbidity, it represents a distinct state of systemic vulnerability. Our adjustment for comorbidity count distinguishes frailty's effects (reduced stress resilience) from direct disease impacts, particularly important given comorbidities' incorporation in ICU severity scores [4,5,22,25,26].

The clinical context of hospitalization category (medical, elective surgical, emergency surgical) reflects fundamental differences in illness acuity and physiological stability [16]. Medical and emergency surgical admissions involve greater physiological instability than optimized elective cases. This adjustment ensures frailty's associations are not confounded by baseline risk differences across admission types [1,2,4,5,16].

Of particular ethical and clinical significance, Life-Sustaining Treatment (LLST) decisions at admission integrate disease severity, prognosis, and patient preferences, fundamentally altering care trajectories and outcomes [1,2,4,5]. By adjusting for LLST, we isolate frailty's prognostic value from treatment limitation effects, crucial for valid interpretation when care intensity varies.

Finally, to capture acute physiological derangements, the 24-hour Sequential Organ Failure Assessment (SOFA) score quantifies acute organ dysfunction, strongly predicting short-term outcomes [22,25]. This adjustment separates acute physiological compromise from chronic vulnerability, aligning with multidimensional evaluation paradigms in critical care [26-28].

This comprehensive adjustment strategy, incorporating variables with strong theoretical and empirical support, enables robust estimation of frailty's independent prognostic value across diverse ICU populations. The simultaneous consideration of acute physiological status and chronic vulnerability represents a significant advancement in critical care outcomes research.
